# A Delphi-based framework for optimizing nurse staffing in Chinese hospitals

**DOI:** 10.3389/fpubh.2025.1510931

**Published:** 2025-07-04

**Authors:** Waner Wang, Gege Li, Jiangfeng Pu, Tiemei Shen, Zhanghao Xie, Lifang Chen, Hong Cui, Peng Xu, Huigen Huang

**Affiliations:** ^1^School of Nursing, Guangdong Pharmaceutical University, Guangzhou, Guangzhou, China; ^2^Department of Nursing, Guangdong Provincial People’s Hospital (Guangdong Academy of Medical Sciences), Southern Medical University, Guangzhou, China; ^3^Jinan University, Guangzhou, China; ^4^Department of Nursing, Shantou University Medical College, Shantou, China; ^5^He You International Hospital Group, Guangzhou, China

**Keywords:** nursing, management, Delphi method, hierarchical analysis, human resource

## Abstract

**Objective:**

The objective of this study was to develop an evaluation system for the allocation of hospital nursing human resources in hospitals in the context of the Healthy China initiative.

**Aims:**

The evaluation system aims to provide a foundation and recommendations for optimizing the allocation of hospital nursing human resources by providing data-driven insights to guide staffing decisions. These recommendations are designed to enhance patient safety, reduce adverse events, and improve overall nursing care quality while supporting the sustainable development of healthcare systems. The system also seeks to enhance nursing human resource management by enabling managers to allocate resources more effectively based on clinical workload, patient needs, and nursing competencies. The primary objectives of this optimization process include reducing nurse workload, improving job satisfaction, and decreasing turnover rates, all of which facilitate improved patient outcomes.

**Methods:**

The evaluation index pool and questionnaire for the index system consultation were developed using a literature review and semi-structured expert interviews. Considering the need for detailed information on the number of experts and the criteria for indicator deletion, we clarified that the study included 26 experts. These experts were selected based on their extensive experience and professional background in nursing management. The criteria for indicator deletion included a variation coefficient of >0.25 and expert consensus on the relevance and importance of the indicators. We conducted two rounds of expert consultations using the Delphi method to screen the evaluation indexes. Subsequently, the weights of the indexes were calculated using the analytic hierarchy process (AHP).

**Results:**

A nursing human resource staffing evaluation system was established, comprising 3 primary indexes, 7 secondary indexes, and 25 tertiary indexes.

**Conclusion:**

The findings of this study provide empirical evidence and specific recommendations for evaluating, guiding, incentivizing, and improving nursing human resource management practices in China. This study is the first structured Delphi-and AHP-based staffing evaluation system in a Chinese hospital setting. By integrating expert consensus and hierarchical analysis, we have developed a novel framework tailored to China’s healthcare needs. This framework significantly contributes to the integrated development of nursing human resource management. Based on the results, we recommend that hospitals implement the evaluation system to regularly monitor and optimize their nursing staffing levels. Moreover, further research may help explore the long-term effects of this system on patient outcomes and nurse retention rates across different hospital settings. Future studies may also examine the adaptability of the evaluation system to various specialties and the potential need for adjustments in specific contexts.

## Introduction

The continuous care provided by nurses is essential for mitigating the risk of clinical accidents and serves as a cornerstone of proactive patient safety measures in healthcare systems. Among high-income regions, California is the first state to implement legislation that mandates comprehensive minimum nurse-to-patient ratios in hospitals. While England has national guidelines in place, the implementation and enforcement are left to local authorities, with no direct legal or economic consequences for failing to adhere to these guidelines. Similarly, Victoria and Queensland (Australia) have adopted mandatory nurse-to-patient ratios, although their methods are more flexible than California’s approach. Ireland’s safety nurse allocation policy involves systematically adjusting the actual staffing level based on the acute needs of patients. Despite having a national framework, the current implementation is limited to pilot sites. Moreover, Japan, South Korea, and Taiwan have developed specific methods for nurse allocation. However, many countries currently lack systematic frameworks or established regulations to define optimal hospital staffing configurations.

Since the early 2000s, nurse staffing levels have been a prominent and contentious issue in healthcare research. Extensive studies have demonstrated that nurse workforce configurations are correlated with severe patient outcomes, including mortality rates (1–3), adverse events (4), and survival rates (5). As a significant proportion of clinical accidents are preventable, monitoring these incidents and ensuring patient safety have become a critical priority garnering substantial attention within the global healthcare community.

In recent years, China has made significant advancements in nursing workforce development. By the end of 2023, China had 5.63 million nurses nationwide, achieving a ratio of 4 nurses per 1,000 people. However, there are challenges such as inconsistencies in nurse staffing across institutions, a lack of standardized evidence-based allocation frameworks, and limited flexibility in adapting to evolving clinical and systemic needs. These issues highlight the urgent need for a scientifically grounded, context-specific staffing evaluation system to guide the rational allocation of nursing resources. Therefore, to address these issues, this study aims to develop a nursing staffing indicator using the Delphi method, which provides specific recommendations for optimizing the allocation of nursing resources in Chinese hospitals.

## The study

### Aims

The objective of this study is to develop an optimal nurse‑staffing system aligned with the objectives of the Healthy China initiative.

### Design

**Figure 1:**
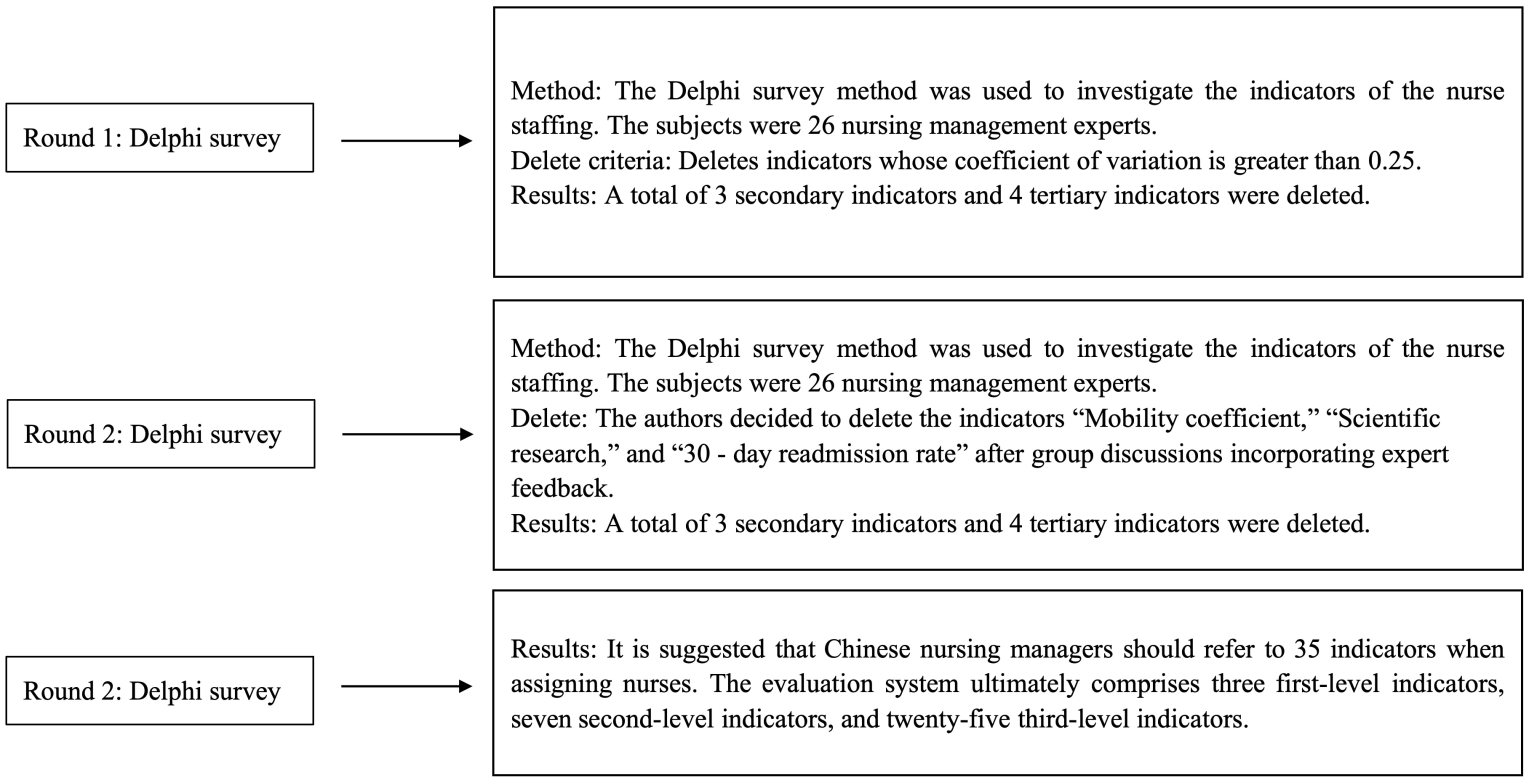
Flowchart of the Delphi.

In the present study, an e-Delphi approach was employed to gather consensus from subject-matter experts (SMEs). The Delphi method uses expert input to systematically develop and prioritize quantitative indicators (6). Initial evaluation indicators were identified after thoroughly reviewing the literature, conducting expert interviews, and discussing with the research team. *A priori* consensus definition was established by the research team, with three criteria per indicator: mean >4.00 and coefficient of variation (CV < 0.25). Once all three criteria were met, consensus was achieved, and further rounds were concluded. The study adhered to the Guidance on Conducting and REporting DElphi Studies (CREDES) (7). Figure 1 illustrates the Delphi process. The theoretical rationale for using the Delphi and Analytic Hierarchy Process (AHP) methods lies in their ability to systematically integrate expert knowledge and prioritize criteria through a structured approach. The Delphi method facilitates consensus-building among nursing and healthcare management experts, ensuring that diverse professional insights are incorporated into the evaluation framework. The AHP provides a structured decision-making model that ranks and weighs staffing criteria based on expert judgment, enabling quantifiable prioritization. The combination of these methods offers a robust foundation for developing a comprehensive set of nurse staffing indicators tailored to the Chinese healthcare context. We employed a Delphi design for this study, forming a structured questionnaire through literature review and semi-structured interviews. This approach reduces the number of consultation rounds and decreases the time required. Many nursing researchers commonly use the Delphi method to achieve thematic consensus (6).

### Participants

Careful selection of experts is crucial in the Delphi method, ensuring that they possess adequate representativeness, authority, and diversity. Factors considered include their professional backgrounds and origins (8). Purposeful sampling strategies were used to recruit participants at every phase of the research, ensuring that they are experienced professionals in their respective fields (9). We selected 26 nursing manager experts from 13 tertiary hospitals across 11 Chinese provinces, namely: Guangdong, Fujian, Liaoning, Xinjiang, Gansu, Yunnan, Guangxi, Chongqing, Jiangxi, Hubei, and Jiangsu, and 1 municipality in Beijing. The criteria for expert selection are as follows: (1) A minimum of 10 years of experience in nursing management and nursing management research; (2) Holding intermediate or higher professional titles; and (3) Actively practicing in areas such as hospital management and management policy research. Experts are needed to meet at least two of the initial three criteria for eligibility (10). Each of these experts possesses extensive experience in nursing management in China and has made significant contributions to the field through their research, as evidenced by their numerous published articles on nursing management. The questionnaires were dispatched to the experts, and they were notified that completing and returning the initial questionnaire signified their willingness to engage in the first survey. Additionally, participants were asked about their willingness to participate in future surveys.

### Data collection

We conducted two rounds of data collection—first via email, then through online surveys administered on the Wenjuanxing platform. The data collection process was completed and presented it for review. The results from each round’s questionnaire were shared with participants in the subsequent round. Participants who did not respond within 10 days received a reminder email, and those who had not responded after 20 days were contacted by phone. All participants were informed that they could withdraw from the process at any time. This survey used a 5-point Likert scale to assess the significance of each indicator. We ensured the security and anonymity of participants by collecting and storing data securely, keeping participants’ identities confidential.

### Questionnaire design

The questionnaire was meticulously designed to collect comprehensive data on nursing staffing indicators. It comprises three main sections: instructions for completion, staffing indicators, and a section for collecting basic information about the experts. A 5-point Likert scale was used to assess the significance of each indicator, with higher ratings indicating greater importance. This scale provides a clear and quantitative method for experts to express their opinions on the relevance and weight of each staffing criterion. Furthermore, a comments section was included to capture experts’ recommendations for modifying calculation formulas, adjusting data collection methods, and including or excluding specific indicators. The questionnaire’s design ensures systematic and structured data collection, allowing the research team to analyze and prioritize the indicators based on expert consensus.

### Ethical considerations

All participants provided their n informed consent. This study was conducted as an e-Delphi consultation and was granted approval from the ethics committee of the Guangdong Provincial People’s Hospital (Approval No. KY2023-806-01, August 2023). Using the results of the literature search and based on the Practical Manual of Nursing Sensitive Quality Indicators (2016 edition) published by the Chinese Centre for Nursing Quality Control, we analyzed and summarized the definition of indicators of nurse staffing. The first draft of the questionnaire was developed based on the initial consensus of all members of our research team. We held an expert formula meeting and invited five experts to comment on the questionnaire. Based on the experts’ comments, the questionnaire was revised and improved. The final questionnaire for nurse staffing consists of three parts: (1) Instructions for filling out the form; (2) Indicators of staffing; and (3) Questionnaire for basic information about experts. Experts utilized a five-point Likert scale to assess the significance of each indicator, where higher ratings denoted higher importance. Additionally, a comment section was creaated to capture experts’ recommendations, including adjustments to calculation formulas and data collection methods, as well as the inclusion or exclusion of indicators.

### Two rounds of expert consultations

On 12 June 2024, the initial e-Delphi survey was conducted via an online questionnaire, followed by the second survey on 16 July 2024, using the same method. Each survey round lasted 1 week. After completing each round, experts were contacted to address any queries regarding their questionnaire responses. Based on the consensus regarding the significance of indicators across the two survey rounds, adjustments, including revision, deletion, or retention, were made to each indicator.

### Data analysis

Data analysis was conducted using Excel 2016 and SPSS 25.0. Descriptive statistics, including measures of central tendency (means) and dispersion (standard deviations), were calculated to summarize the measurement data. Additionally, categorical variables were represented as frequencies to provide a more comprehensive overview of data distribution. Inferential statistical tests, such as chi-squared tests for categorical variables and t-tests for continuous variables, were performed, as appropriate, to examine the significance of differences and associations between variables. Expert positivity was quantified as the effective rate of the questionnaire. The expert authority coefficient (Cr) was calculated to assess the reliability and credibility of the expert panel. Derived from judgment grounds (Cs) and familiarity level (Ca), the Cr value was determined to be 0.869, indicating a high level of authority and consistency among the experts’ responses. This coefficient was calculated using the formula Cr = (Cs + Ca)/2, where Cs reflects the experts’ judgment basis and Ca reflects their familiarity with the topic. The variation and Kendall coefficients of concordance (W) were computed to evaluate the degree of coordination among the experts’ opinions. Kendall’s W value was found to be 0.78, indicating a high level of agreement among the experts. The mean importance and variation coefficients were analyzed to gauge the consistency of the expert advice. The AHP was utilized with Yaahp 10.3 software to calculate the indicator weights, combined weights, and consistency ratio (CR). The CR value was 0.08, which is within the acceptable threshold of less than 0.1, ensuring the reliability of the prioritization process. Statistical significance for all tests was established at a *p*-value of <0.05 (Table 1).

**Table 1:** Degree of expert authority (*n* = 26).

Expert number	Coefficient of judgement (Ca)	Familiarity (Cs)	Authority factor (Cr)
S1	0.8	0.8	0.8
S2	1	1	1
S3	0.95	1	0.975
S4	0.8	0.8	0.8
S5	0.9	0.8	0.85
S6	0.9	0.8	0.85
S7	0.8	0.8	0.8
S8	0.8	1	0.9
S9	0.8	0.8	0.8
S10	0.65	0.8	0.725
S11	0.9	1	0.95
S12	0.85	1	0.925
S13	1	1	1
S14	1	1	1
S15	0.7	0.6	0.65
S16	0.7	0.6	0.65
S17	0.65	0.8	0.725
S18	1	1	1
S19	0.8	0.8	0.8
S20	0.8	0.8	0.8
S21	0.4	1	0.7
S22	1	1	1
S23	1	1	1
S24	0.8	1	0.9
S25	1	1	1
S26	1	1	1

## Results

### Expert characteristics and correlation coefficients

In the initial phase of the investigation, we distributed 26 human resources survey questionnaires and received responses from all participants, resulting in a 100% response rate and significant expert engagement. This 100% response rate suggests a high level of commitment and involvement from the experts, which enhances the validity and robustness of our findings. The experts had diverse educational backgrounds, with 3 holding doctoral degrees, 6 holding master’s degrees, and 17 holding undergraduate degrees. Furthermore, 15 experts held full senior titles, 10 experts held associate senior titles, and 1 expert held an intermediate title. The Kendall’s coefficient of concordance (W) was 0.78, which was statistically significant (*p* < 0.05), as determined by the chi-squared test. This significant Kendall’s W value indicates a high degree of agreement among experts’ opinions, reinforcing the reliability of the study’s findings (Table 2 presents the coefficient of authority of experts involved in this study).

**Table 2:** Degree of harmonization of expert opinions.

Range of indicator fluctuations	Equipped with indicators (secondary indicators)		Equipped with indicators (Tertiary indicators)	
	Round 1	Round 2	Round 1	Round 2
Kendall’s *W*	0.217	0.073	0.135	0.118
$x^{2}$	107.054	11.346	199.843	73.673
*p*	<0.001	0.078	<0.001	<0.001

### Indicator modifications

The study initially considered 38 indicators. After collective deliberation among authors and feedback from experts, a set of 25 tertiary indicators, 7 secondary indicators, and 3 primary indicators was retained for nurse staffing. The authors decided to remove the indicators “Mobility coefficient,” “Scientific research,” and “30-day readmission rate” following group discussions that incorporated expert feedback. The weights of the indicators are summarized in Table 3, with the highest weights assigned to “structure of nursing positions” and “staffing.” During the indicator selection process, criteria such as a coefficient of variation of >0.25 were applied to ensure the reliability and validity of the results.

**Table 3:** Delphi first round inquiry item increase and decrease situation.

Content		First round inquiry			
		Original number of items	Increase number of items	Decrease number of items	Removed items
Secondary indicator	Structure	3	0	1	Nursing Position Structure
Secondary indicator	Process	4	0	2	Staffing, Nursing Work Range
Tertiary indicator	Nursing Workload	3	0	1	DRG Grouping
Tertiary indicator	Staffing	3	0	1	Mechanism
Tertiary indicator	Nursing Work Range	3	0	1	Scientific Research Work
Tertiary indicator	Nursing Quality	3	0	1	(30-day) Readmission Rate

## Discussion

This study uses the Delphi expert consultation and hierarchical analysis methods to establish a nurse staffing evaluation system. Specifically, this evaluation system comprises 3 primary indicators, 7 secondary indicators, and 25 tertiary indicators. The credibility of this evaluation system is enhanced by extensive reviews of literature and policy documents and validated through multiple expert validation meetings, semi-structured interviews, and iterative discussions to establish a preliminary indicator repository. Experts were selected based on their educational backgrounds and professional experiences, resulting in a panel of 26 individuals with robust theoretical knowledge and practical expertise in nursing management. These experts demonstrated high authority and received positive coefficients in their evaluations. The judgment matrix used in the hierarchical analysis passed consistency tests, ensuring reliable consultation outcomes. This robust process provides a strong foundation for evaluating nursing staffing (Table 4).

**Table 4:** Second round of expert consultation item reduction situation.

Content		Second round			Final number of items formed
		Original number of items	Number of items added	Decrease number of items	
Indicators	Secondary indicator	7	0	0	7
	Tertiary indicator	25	0	0	25

The nurse staffing evaluation system consists of 3 primary indicators, 7 secondary indicators, and 25 tertiary indicators. Among the secondary indicators, nurse allocation carries the highest weight at 0.222. Consistent with relevant research results, a reasonable allocation of nursing human resources is conducive to nurses caring for patients. When nurses take care of one less patient, the adverse report of nursing quality is reduced by 1.04 times (11). Followed by nurses’ core competence at 0.214, experts prioritize the core competence of nurses, aligning with existing research findings. As frontline clinical personnel, nurses’ professional knowledge and skills significantly impact the quality and safety of nursing care, thereby making it a critical determinant for the allocation of nursing human resources. This underscores the pivotal role of nurses’ core competence in enhancing medical service capabilities and hospital cultural influence. Therefore, it is imperative to use both the internal values and external behaviors of nurses when managing nursing personnel in hospitals. The top four tertiary indicators are ranked as follows: nurse-to-patient ratio holds the highest weight of 0.167, followed by adverse event incidence (0.120), nursing workload (0.0962), and emergency response capability (0.0712). The National Nursing Career Development Plan (2016–2020) (12) explicitly emphasizes indicators such as nurses per 1,000 individuals and bed-to-nurse ratio as fundamental metrics for nursing allocation strategies. Therefore, future efforts in nursing management should prioritize nurse staffing to enhance patient safety; it is recommended to adhere to the “quality first” management principle to reduce adverse events. Implementing this approach involves systematic and meticulous patient safety management across all operational aspects. Moreover, the capability for emergency treatment should not be overlooked. Ensuring effective and rapid emergency response in case of changes in patient conditions or accidents is crucial for safeguarding patient lives. Finally, nursing intensity should be carefully considered. In daily work organization, nurses should be allocated flexibly based on anticipated work intensity, to maintain a balanced level of nursing intensity (13). The nurse-to-patient ratio correlates with patient safety. A longitudinal study evaluating nurse staffing levels and hospitalization rates in Michigan (2003–2006) revealed that each nurse-to-patient ratio change corresponded to a mortality reduction rate of 0.25% (14). This change in ratio affects nurses’ work efficiency and job stability. Imbalanced ratios can overload nurses, compromising patient safety and causing job instability. A 2022–2023 survey of 9,150 registered nurses showed that 29.4% planned to leave their jobs due to high workloads. Suboptimal staffing configurations can lead to nurse burnout, increased turnover, and weakened response capabilities (15). When nurses handle more patients than the recommended threshold, the risk of medication errors and delayed illness identification increases (Table 5).

**Table 5:** Summary of the results of the evaluation of the indicator system.

Primary indicators	Rating ( $\bar{x}\pm s$ )	Weights	Secondary indicators	Rating ( $\bar{x}\pm s$ )	weights	Tertiary indicators	Rating ( $\bar{x}\pm s$ )	Weights
1. Structure	5.00 ± 0.00	0.3333	1.1 Number of nurses staffed	4.85 ± 0.464	0.2222	1.1.1 Bed-guard ratio	4.65 ± 0.562	0.0556
						1.1.2 Nurse–patient ratio	4.92 ± 0.272	0.1667
			1.2 Nurse structure configuration	4.65 ± 0.562	0.1111	1.2.1 Percentage of nurses with different academic qualifications	4.38 ± 0.752	0.0278
						1.2.2 Percentage of nurses with different years of service	4.58 ± 0.578	0.0556
						1.2.3 Percentage of nurses with different job titles	4.38 ± 0.752	0.0278
						1.2.3 Percentage of specialist nurses	4.65 ± 0.562	0.0112
						1.2.4 Percentage of Clinical Nursing Positions	4.81 ± 0.402	0.0114
						1.2.5 Percentage of nursing management positions	4.58 ± 0.504	0.0075
2. Process	5.00 ± 0.00	0.3333	2.1 Nursing workload	4.73 ± 0.452	0.1111	2.1.1 Intensity of care	4.85 ± 0.368	0.0239
						2.1.2 Proportion of levels of care	4.62 ± 0.637	0.0114
						2.1.3 Annual leave days	4.46 ± 0.761	0.0064
						2.1.4 Rest coefficient	4.46 ± 0.761	0.0125
						2.1.5 Training Leads	4.69 ± 0.471	0.0174
						2.1.6 Continuity of care	4.46 ± 0.647	0.0064
			2.2 Core competencies for nurses	4.85 ± 0.368	0.2222	2.2.1 Competence in clinical practice	4.81 ± 0.402	0.0691
						2.2.2 Emergency response capacity	4.85 ± 0.368	0.1096
						2.2.3 Communication and education capacity	4.69 ± 0.471	0.0435
3. Results	5.00 ± 0.00	0.3333	3.1 Volume of nursing services	4.77 ± 0.430	0.0990	3.1.1 Bed occupancy rate	4.81 ± 0.402	0.0495
						3.1.2 Average length of stay	4.73 ± 0.452	0.0247
						3.1.3 Hours of 24-h care per inpatient stay	4.73 ± 0.533	0.0247
			3.2 Quality of care	4.92 ± 0.272	0.1799	3.2.1 Incidence of adverse events	4.73 ± 0.533	0.1199
						3.2.2 Mortality	4.62 ± 0.752	0.0600
			3.3 Satisfaction evaluation	4.62 ± 0.571	0.0545	3.3.1 Patient satisfaction	4.73 ± 0.452	0.0162
						3.3.2 Nurse satisfaction	4.88 ± 0.326	0.0294
						3.3.3 Nurse Separation Rate	4.58 ± 0.643	0.0089

## Limitations

Due to time constraints, limited experience, and practical considerations, the selected sources of experts are somewhat restricted, which may affect the comprehensiveness of their representation. Therefore, it is advisable to broaden the scope of experts included. Notably, the developed evaluation system may primarily apply to tertiary hospitals and might not be directly suitable for community or rural healthcare institutions. Rural and community medical institutions are characterized by distinct attributes, including extensive service radii, fragmented patient needs, substantial chronic disease management burdens, specific service modalities, and unique team compositions. Empirical evaluation of the existing nursing manpower allocation framework in these primary care settings is imperative to systematically assess its adaptability. This study advances the theoretical generalizability of nursing workforce allocation models and enhances the quality and accessibility of healthcare services in underserved rural and community contexts.

## Conclusion

This study used the Delphi method and AHP to develop a scientifically grounded evaluation system for the allocation of nursing human resources in Chinese hospitals. After two rounds of expert consultation, an initial set of 38 indicators was refined to a final framework comprising 35 indicators: 3 first-level indicators, 7 second-level indicators, and 25 third-level indicators. This reduction reflects expert consensus on the most relevant and practical measures, enhancing the system’s applicability in real-world settings. The most impactful indicators identified were the nurse-to-patient ratio, workload intensity, and alignment between nurse qualifications and job requirements, directly influencing service quality and patient safety. The structured framework can support hospital administrators in making more informed, data-driven decisions regarding staffing distribution, which improves efficiency and outcomes across clinical departments. This study provides a foundation for standardizing the evaluation of nurse staffing in China and contributes to the broader objective of enhancing healthcare system performance. However, there are limitations including the relatively small size of the expert panel and the potential for subjective bias in the weighting of indicators. Future research should focus on validating this framework across diverse hospital types and regions and exploring the integration of real-time data and AI tools into staffing decision systems. The proposed indicator system offers practical guidance for optimizing nursing resource management and aligns with national health policy goals, thereby fostering the strategic development of the nursing workforce in China.

## Data Availability

The raw data supporting the conclusions of this article will be made available by the authors, without undue reservation.

## Author’s note

The indicators identified in this study are pragmatic and closely linked to clinical management. These indicators were developed based on various studies and expert opinions, offering valuable insights into management decision-making.
