# Supplementary material for: A Delphi-based framework for optimizing nurse staffing in Chinese hospitals
Source: Front Public Health. 2025 Jul 4;13:1510931. doi: 10.3389/fpubh.2025.1510931 (PMC12271208; doi:10.3389/fpubh.2025.1510931)
Supplement: Supplementary file 1 [file Data_Sheet_1.pdf]

Table 1 Degree of expert authority (n=26)

Expert number	Coefficient of judgement (Ca)	Familiarity (Cs)	Authority factor (Cr)
S1	0.8	0.8	0.8
S2	1	1	1
S3	0.95	1	0.975
S4	0.8	0.8	0.8
S5	0.9	0.8	0.85
S6	0.9	0.8	0.85
S7	0.8	0.8	0.8
S8	0.8	1	0.9
S9	0.8	0.8	0.8
S10	0.65	0.8	0.725
S11	0.9	1	0.95
S12	0.85	1	0.925
S13	1	1	1
S14	1	1	1
S15	0.7	0.6	0.65
S16	0.7	0.6	0.65
S17	0.65	0.8	0.725
S18	1	1	1
S19	0.8	0.8	0.8
S20	0.8	0.8	0.8
S21	0.4	1	0.7
S22	1	1	1
S23	1	1	1
S24	0.8	1	0.9
S25	1	1	1
S26	1	1	1

Table 2 Degree of harmonization of expert opinions

Range of indicator fluctuations	Equipped with indicators (secondary indicators)		Equipped with indicators (Tertiary indicators)	
	Round 1	Round 2	Round 1	Round 2
Kendall's W	0.217	0.073	0.135	0.118
x^2	107.054	11.346	199.843	73.673
P	<0.001	0.078	<0.001	<0.001

Table 3 Delphi First Round Inquiry Item Increase and Decrease Situation

content		First Round Inquiry			
		Original Number of Items	Increase Number of Items	Decrease Number of Items	Removed Items
Secondary indicator	Structure	3	0	1	Nursing Position Structure
Secondary indicator	Process	4	0	2	Staffing, Nursing Work Range
Tertiary indicator	Nursing Workload	3	0	1	DRG Grouping
Tertiary indicator	Staffing	3	0	1	Mechanism
Tertiary indicator	Nursing Work Range	3	0	1	Scientific Research Work
Tertiary indicator	Nursing Quality	3	0	1	(30-day) Readmission Rate

Table 4 Second Round of Expert Consultantion Item Reduction Situation

content		Second Round			Final Number of Items Formed
		Original Number of Items	Number of Items Added	Decrease Number of Items	
indicators	Secondary indicator	7	0	0	7
	Tertiary indicator	25	0	0	25

Table 5 Summary of the results of the evaluation of the indicator system

Primary indicators	Rating ($\bar{x} \pm s$)	weights	Secondary indicators	Rating ($\bar{x} \pm s$)	weights	Tertiary indicators	Rating ($\bar{x} \pm s$)	weights
1. Structure	5.00± 0.00	0.3333	1.1 Number of nurses staffed	4.85± 0.464	0.2222	1.1.1 Bed-guard ratio	4.65± 0.562	0.0556
						1.1.2 Nurse-patient ratio	4.92± 0.272	0.1667
			1.2 Nurse structure configuration	4.65± 0.562	0.1111	1.2.1 Percentage of nurses with different academic qualifications	4.38± 0.752	0.0278
						1.2.2 Percentage of nurses with different years of service	4.58± 0.578	0.0556
						1.2.3 Percentage of nurses with different job titles	4.38± 0.752	0.0278
						1.2.3 Percentage of specialist nurses	4.65± 0.562	0.0112

						1.2.4 Percentage of Clinical Nursing Positions	4.81± 0.402	0.0114
						1.2.5 Percentage of nursing management positions	4.58± 0.504	0.0075
2. Process	5.00± 0.00	0.3333	2.1 Nursing workload	4.73± 0.452	0.1111	2.1.1 Intensity of care	4.85± 0.368	0.0239
						2.1.2 Proportion of levels of care	4.62± 0.637	0.0114
						2.1.3 Annual leave days	4.46± 0.761	0.0064
						2.1.4 Rest coefficient	4.46± 0.761	0.0125
						2.1.5 Training Leads	4.69± 0.471	0.0174
						2.1.6 Continuity of care	4.46± 0.647	0.0064
			2.2 Core competencies for nurses	4.85± 0.368	0.2222	2.2.1 Competence in clinical practice	4.81± 0.402	0.0691
						2.2.2 Emergency response capacity	4.85± 0.368	0.1096

						2.2.3 Communication and education capacity	4.69± 0.471	0.0435
3. Results	5.00± 0.00	0.3333	3.1 Volume of nursing services	4.77± 0.430	0.0990	3.1.1 Bed occupancy rate	4.81± 0.402	0.0495
						3.1.2 Average length of stay	4.73± 0.452	0.0247
						3.1.3 Hours of 24-hour care per inpatient stay	4.73± 0.533	0.0247
			3.2 Quality of care	4.92± 0.272	0.1799	3.2.1 Incidence of adverse events	4.73± 0.533	0.1199
						3.2.2 Mortality	4.62± 0.752	0.0600
			3.3 Satisfaction evaluation	4.62± 0.571	0.0545	3.3.1 Patient satisfaction	4.73± 0.452	0.0162
						3.3.2 Nurse satisfaction	4.88± 0.326	0.0294
						3.3.3 Nurse Separation Rate	4.58± 0.643	0.0089
