# Supplementary material for: A Delphi-based framework for optimizing nurse staffing in Chinese hospitals
Source: Front Public Health. 2025 Jul 4;13:1510931. doi: 10.3389/fpubh.2025.1510931 (PMC12271208; doi:10.3389/fpubh.2025.1510931)
Supplement: Supplementary file 2 [file Data_Sheet_2.docx]

Nursing Human Resource Allocation Expert Questionnaire - First Round

Dear Expert,

Greetings! Thank you for taking the time out of your busy schedule to review this questionnaire. We sincerely invite you to serve as a consulting expert for the "Study on Nursing Human Resource Allocation Standards" under the China Health Talent Training Project. Your opinions on this plan directly affect its practicality and scientific nature. Please evaluate the relevant indicators of nursing human resource allocation in the following questionnaire and provide suggestions based on your extensive experience in nursing management.

This study requires two rounds of expert consultation, and this is the first round. Each round will take approximately 25-30 minutes of your valuable time. All members of our team express their heartfelt gratitude for your contribution! We assure you that your personal information and opinions will be kept completely confidential!

The nursing human resource allocation system consulted this time is divided into three parts: 3 first-level indicators, 7 second-level indicators, and 30 third-level indicators, derived from questionnaire surveys, expert interviews, and expert meetings. Please provide your valuable comments based on your understanding of each option in the system.

Due to tight timing, we sincerely hope you can return it to the email by June 19, 2024.

For inquiries, please call: Research team member Wang XX 150880XXXX

Email: [2212453260@qq.com](mailto:2212453260@qq.com)

Address: No. 106 Zhongshan Second Road, Yuexiu District, Guangzhou City, Guangdong Province, XXXX Hospital

Thank you again for your help amidst your busy schedule, and we wish you smooth work and good health!

The Research Team of "Study on Nursing Human Resource Allocation Standards"

Allocation Indicators

Main Content: Indicators for Nursing Human Resource Allocation

Instructions for filling out the form:

The allocation indicators are derived from the project team through expert interviews and expert meetings.

Please rate the specific indicators listed, and a column ("Indicators you think need to be added") has been reserved at the end for you to enter indicators that you believe should be included but are not listed, and rate them accordingly. Please do not leave any items blank or missed, thank you!

Table 1 Judgment of Secondary Indicators

| 1.1 Judgment of Secondary Indicators  ►Please mark "√" to evaluate the importance, feasibility, and effectiveness of the secondary indicators, and provide your valuable suggestions for modifications. | | | | | | | |
| --- | --- | --- | --- | --- | --- | --- | --- |
| First-level Indicator | Secondary Indicator | Importance | | | | | Suggestions and Comments |
|  |  | Very Important | Important | Moderately Important | Not Very Important | Not Important |  |
|  |  | 5 | 4 | 3 | 2 | 1 |  |
| 1 Structure | 1.1 Staffing of Nurses |  |  |  |  |  |  |
|  | 1.2 Configuration of Nurse Structure |  |  |  |  |  |  |
|  | 1.3 Structure of Nursing Positions |  |  |  |  |  |  |
| 2 Process | 2.1 Nursing Workload |  |  |  |  |  |  |
|  | 2.2 Staffing |  |  |  |  |  |  |
|  | 2.3 Scope of Nursing Work |  |  |  |  |  |  |
|  | 2.4 Core Competencies of Nurses |  |  |  |  |  |  |
| 3 Results | 3.1 Volume of Nursing Services |  |  |  |  |  |  |
|  | 3.2 Quality of Care |  |  |  |  |  |  |
|  | 3.3 Satisfaction Evaluation |  |  |  |  |  |  |

Tertiary Indicators

Table 2 Judgment of Tertiary Indicators

| Please mark "√" to evaluate the importance, feasibility, and effectiveness of the tertiary indicators, and provide your valuable suggestions for modifications. | | | | | | | | |
| --- | --- | --- | --- | --- | --- | --- | --- | --- |
| Indicator | | | Importance | | | | | Suggestions and Comments |
| First-level Indicator | Secondary Indicator | Tertiary indicators |  |  |  |  |  |  |
|  |  |  | very important | important | moderate | Not Important | very unimportant |  |
|  |  |  | 5 | 4 | 3 | 2 | 1 |  |
| 1Structure | 1.1 Staffing of Nurses | 1.1.1 Nurse-to-patient Ratio |  |  |  |  |  |  |
|  |  | 1.1.2 Bed-to-Nurse Ratio |  |  |  |  |  |  |
|  | 1.2 Configuration of Nurse Structure | 1.2.1 Proportion of Nurses with Different Academic Qualifications |  |  |  |  |  |  |
|  |  | 1.2.2 Proportion of Nurses with Different Years of Service |  |  |  |  |  |  |
|  |  | 1.2.3 Proportion of Nurses with Different Job Titles |  |  |  |  |  |  |
|  | 1.3 Structure of Nursing Positions | 1.3.1 Proportion of Specialist Nurses |  |  |  |  |  |  |
|  |  | 1.3.2 Proportion of Clinical Nursing Positions |  |  |  |  |  |  |
|  |  | 1.3.3 Proportion of Nursing Management Positions |  |  |  |  |  |  |
| 2 Process | 2.1 Nursing Workload | 2.1.1Intensity of Nursing Care |  |  |  |  |  |  |
|  |  | 2.1.2 DRG Grouping |  |  |  |  |  |  |
|  |  | 2.1.3 Proportion of Nursing Levels |  |  |  |  |  |  |
|  | 2.2 Staffing | 2.2.1 Annual leave Days |  |  |  |  |  |  |
|  |  | 2.2.2 Rest Coefficient |  |  |  |  |  |  |
|  |  | 2.2.3 Scope of Work |  |  |  |  |  |  |
|  | 2.3 Scope of Work | 2.3.1 Training and Teaching |  |  |  |  |  |  |
|  |  | 2.3.2 Scientific Research Work |  |  |  |  |  |  |
|  |  | 2.3.3 Continuous Nursing |  |  |  |  |  |  |
|  | 2.4 Core Competencies of Nurses | 2.4.1 Clinical Practice Ability |  |  |  |  |  |  |
|  |  | 2.4.2 Emergency Response Ability |  |  |  |  |  |  |
|  |  | 2.4.3 Communication and Education Ability |  |  |  |  |  |  |
| 3 Results | 3.1 Volume of Nursing Services | 3.1.1 Bed Occupancy Rate |  |  |  |  |  |  |
|  |  | 3.1.2 Average Length of Stay |  |  |  |  |  |  |
|  |  | 3.1.3 Hours of 24-hour Care per Inpatient Stay |  |  |  |  |  |  |
|  | 3.2 Quality of Care | 3.2.1 Incidence of Adverse Events |  |  |  |  |  |  |
|  |  | 3.2.2 Mortality Rate |  |  |  |  |  |  |
|  |  | 3.2.3 (30-day) Readmission Rate |  |  |  |  |  |  |
|  | 3.3 Satisfaction Evaluation | 3.3.2 Patient Satisfaction |  |  |  |  |  |  |
|  |  | 3.3.1 Nurse Satisfaction |  |  |  |  |  |  |
|  |  | 3.3.3 Nurse Turnover Rate |  |  |  |  |  |  |

**Explanations:**

1. Bed-to-Nurse Ratio: The ratio of the number of actually open beds to the number of practicing nurses in a medical institution within a unit of time.
2. Nurse-to-Patient Ratio: The ratio of the total number of responsible nurses per day to the total number of inpatients under their care within a unit of time.
3. Nursing Intensity: The workload completed by nurses within a certain period, including weekly working days, daily working hours, monthly night shift frequency, and perceptions of night shifts, etc.
4. DRG Grouping: Diagnosis Related Groups (DRG) is an important tool for measuring the quality and efficiency of medical services and for medical insurance payment. Essentially, DRG is a case-mix classification scheme that categorizes patients into several diagnostic groups for management based on factors such as age, disease diagnosis, comorbidities, complications, treatment methods, severity of illness, outcomes, and resource consumption.
5. Nursing Care Level Proportion: Reflects the urgency and severity of patients' conditions, as well as their nursing needs and workload. It helps managers estimate nursing work load and serves as an important basis for the rational allocation of nursing human resources.
6. Rest Coefficient (CR): A workload coefficient added based on measured working hours requirements. Rest Coefficient = 365 / (365 - number of rest days).
7. Mobility Coefficient: Refers to the additional staff members added to the general staffing number to account for situations such as vacations, maternity leave, external training, sick leave, etc.
8. Core Competency: The special knowledge, skills, judgment, and personal attributes required of nurses to provide safe and ethically compliant nursing services.
9. Bed Utilization Rate: Reflects the ratio of daily occupied beds to the total available beds, i.e., the ratio of total bed-days actually occupied to total bed-days actually open.
10. Average Length of Stay (ALOS): The ratio of total bed-days occupied by discharged patients to the number of discharged patients.
11. Nursing Hours per Inpatient per 24 Hours: The ratio of the actual working hours of practicing nurses in a medical institution's ward to the total bed-days occupied by inpatients during the statistical period. 12. Incidence Rate of Adverse Events: Refers to safety hazards, states, or negative events with consequences caused by factors other than the natural progression of the patient's own disease, which are actively discovered by staff in the hospital or occur during the patient's diagnosis and treatment.
12. Mortality Rate: Represents the proportion of people or animals that die from a specific disease within a certain period relative to the total number of people or animals with the disease.
13. Readmission Rate: The proportion of patients who are readmitted within 30 days of discharge for any reason (excluding planned readmissions) to the total number of discharged patients within the same period.
14. Patient Satisfaction: The degree of consistency between patients' expectations of medical services and their actual perception of those services.
15. Nurse Satisfaction: Nurses' overall attitude toward their work, reflecting the degree to which their needs are met.

17. Nurse Turnover Rate: The ratio of the number of nurses leaving a medical institution to the total number of practicing nurses within a unit of time.

Expert Information Main Content: Basic Information of Experts, Expert Authority Information Instructions for Filling out the Form: Please fill in the following content according to your own situation or mark "√" in the corresponding items.

**Basic Information of Experts**

1. Your Name: ________

2. Your Hospital/Institution: ________

3. Hospital Level: □ Secondary □ Tertiary

4. Gender: □ Male □ Female

5. Age: ______ years old

6. Years of Work Experience: ______ years

7. Work Field: □ Nursing Management □ Nursing Education □ Clinical Nursing □ Medical □ Other

8. Highest Academic Degree: □ Junior College or Below □ Bachelor’s Degree □ Master’s Degree □ Doctoral Degree □ Other ___

9. Administrative Position: ____________________ (please specify)

10. Professional Title: □ Primary Professional Title □ Intermediate Professional Title □ Associate Senior Professional Title □ Senior Professional Title 11. Have you had experience in nursing human resources management? □ Yes □ No

1. Expert Authority Information
2. Please indicate your level of familiarity with the research question Instructions: Please mark “√” in the corresponding position according to your actual situation. □ Very familiar 0.9  □ Relatively familiar 0.7  □ Moderately familiar 0.5  □ Somewhat unfamiliar 0.3  □ Very unfamiliar 0.1

2. Basis for Expert Judgment Instructions: The following four aspects may influence your judgment on this question. The degree of influence of each aspect on your judgment is divided into three levels: high, medium, and low. Please mark “√” in the corresponding position according to your own situation.

| Basis for Judgment | Impact level | | |
| --- | --- | --- | --- |
|  | High | Medium | Low |
| Theoretical Analysis | 0.3 | 0.2 | 0.1 |
| Practical Experience | 0.5 | 0.4 | 0.3 |
| Understanding of Domestic and International Situations | 0.1 | 0.1 | 0.1 |
| Intuition | 0.1 | 0.1 | 0.1 |

**Thanks again for your help.**

# **Second-Round Expert Questionnaire on Nursing Human Resources Allocation**

# Dear Expert,

# Greetings! This project is one of the sub-projects of the Nursing Research under the China Health Talent Development Program, led by XXX (XXXXX Hospital). Thank you for participating in the first-round expert consultation and providing valuable insights. We are now sharing the statistical results from the previous round with all experts. Please complete this second-round questionnaire based on these results.

# Instructions for Filling out the

# Questionnaire:1. The questionnaire is divided into four parts: Part 1: Allocation Principles; Part 2: Allocation Factors; Part 3: Allocation Indicators; Part 4: Nurse-to-Patient Ratios for Secondary and Tertiary Hospitals. 2. The statistical analysis results and expert feedback from the previous round are included in the stem of each question. Please read them carefully before answering. 3. If you need to modify, delete, or add indicators, please note your revisions in the "Modification Suggestions" column.

# Address: No. XX, XX Road, Yuexiu District, Guangzhou City, Guangdong Province, XXXXX Hospital

# Contact Number: 1803896XXXX (Ms. Wang)

# Thank you for your strong support for this project! We wish you smooth work and good health.

# If you have any questions or suggestions about our research, please feel free to contact us.

# 1. Your Name: [Fill in the blank] *_________________________________

# 2. Your Hospital/Institution: [Fill in the blank] ____________________

Part I: Equipped with indicators

**Secondary indicators**

1. This part discusses the secondary indicators of the nursing human resources staffing indicators, with a total of 10 entries. In the previous round, the average score of each entry in this part was 4.84, the coefficient of variation of importance ranged from 0.040 to 0.138, and the coefficient of variation of feasibility ranged from 0.141 to 0.212. According to the criteria for selecting the indicators formulated by the research group, it is necessary to delete the following entries: ‘Nursing post structure’, ‘Staffing’, and ‘Nursing work scope’. According to the criteria for the selection of indicators proposed by this group, it is necessary to delete ‘nursing position structure’, ‘staffing’ and ‘nursing scope of work’, so please rate the importance of the remaining entries.

2. Level of importance* *

|  | Very Important | Important | Moderate | not important | very Unimportant |
| --- | --- | --- | --- | --- | --- |
| 1 Nurse quantity configuration | ○ | ○ | ○ | ○ | ○ |
| 2 Nurse structure configuuration | ○ | ○ | ○ | ○ | ○ |
| 3 Nursing workload | ○ | ○ | ○ | ○ | ○ |
| 4 Nurse core competence | ○ | ○ | ○ | ○ | ○ |
| 5 Nursing service volume | ○ | ○ | ○ | ○ | ○ |
| 6 Nursing quality | ○ | ○ | ○ | ○ | ○ |
| 7 Satisfaction evaluation | ○ | ○ | ○ | ○ | ○ |

3.Revision suggestions

_________________________________

**Tertiary Indicators**

1. This section discusses the tertiary indicators of nursing human resource allocation, totaling 17 items. In the previous round, the average score for each item in this section was 4.69, with a range of variation coefficients for importance from 0.040 to 0.204, and for feasibility from 0.113 to 0.252. According to the selection criteria for indicators established by our research team, the items "DRG Grouping," "Mobility Coefficient," "Scientific Research Work," and "30-day Readmission Rate" need to be removed. Please rate the importance and feasibility of the remaining items.

2. Importance of Tertiary Indicators [Matrix Single-Choice Question] *

Explanations:

1. Bed-to-Nurse Ratio: The ratio of the actual number of open beds in a medical institution to the number of practicing nurses within a unit of time.

2. Nurse-to-Patient Ratio: The ratio of the sum of the number of responsible nurses each day to the sum of the number of inpatients they are responsible for within a unit of time.

3. Nursing Intensity: The amount of work a nurse completes within a certain period of time, including the number of working days per week, daily working hours, monthly night shift frequency, and feelings about night shifts.

4. Nursing Level Ratio: Reflects the urgency and severity of patients' conditions, as well as their nursing needs and workload, which can help managers estimate the nursing workload and is an important basis for the reasonable arrangement of nursing human resources.

6. Rest Coefficient: Abbreviated as CR, it is a coefficient added based on the requirements of measured working hours. Rest Coefficient = 365 / (365 - number of rest days).

7. Core Competencies: The special knowledge, skills, judgment, and personal qualities required of nurses to provide safe and ethical nursing care.

8. Bed Utilization Rate: Reflects the ratio of beds used each day to the actual number of beds, i.e., the ratio of the total number of occupied bed days to the total number of open bed days.

9. Average Length of Stay: The ratio of "total bed days occupied by discharges" to "number of discharges."

10. Hours of 24-hour Nursing per Inpatient Stay: The ratio of the actual working hours of practicing nurses in the medical institution's ward to the actual number of bed days occupied by inpatients during the statistical period.

11. Incidence of Adverse Events: Refers to the negative events caused by various factors other than the natural course of the patient's disease, which are actively discovered by staff or occur during the patient's medical treatment.

12. Mortality Rate: Indicates the proportion of people or animals that died from a certain disease within a certain period to the total number of people or animals with the disease.

14. Patient Satisfaction: The degree of consistency between patients' expectations for medical services and their actual perception of medical services.

15. Nurse Satisfaction: The overall attitude of nurses towards their work, which reflects the degree to which their needs are met.

16. Nurse Turnover Rate: The ratio of the number of nurses who leave a medical institution to the total number of practicing nurses within a unit of time.

|  | Very Important | Important | Moderate | not important | very Unimportant |
| --- | --- | --- | --- | --- | --- |
| 1. Nurse-to-Patient Ratio | ○ | ○ | ○ | ○ | ○ |
| 2. Bed-to-Nurse Ratio | ○ | ○ | ○ | ○ | ○ |
| 3. Proportion of Nurses with Different Academic Backgrounds | ○ | ○ | ○ | ○ | ○ |
| 4. Proportion of Nurses with Different Years of Service | ○ | ○ | ○ | ○ | ○ |
| 5. Proportion of Nurses with Different Job Titles | ○ | ○ | ○ | ○ | ○ |
| 6. Proportion of Specialist Nurses | ○ | ○ | ○ | ○ | ○ |
| 7. Proportion of Clinical Nursing Positions | ○ | ○ | ○ | ○ | ○ |
| 8. Proportion of Nursing Management Positions | ○ | ○ | ○ | ○ | ○ |
| 9.Nursing Intensity | ○ | ○ | ○ | ○ | ○ |
| 10. Proportion of Nursing Shifts | ○ | ○ | ○ | ○ | ○ |
| 11.Number of Annual Leave Days | ○ | ○ | ○ | ○ | ○ |
| 12. Rest Coefficient | ○ | ○ | ○ | ○ | ○ |
| 13. Training Load | ○ | ○ | ○ | ○ | ○ |
| 14. Continuous Nursing Care | ○ | ○ | ○ | ○ | ○ |
| 15. Clinical Practice Ability | ○ | ○ | ○ | ○ | ○ |
| 16.Emergency Response Capability | ○ | ○ | ○ | ○ | ○ |
| 17.Communication and Education Ability | ○ | ○ | ○ | ○ | ○ |
| 18. Bed Utilization Rate | ○ | ○ | ○ | ○ | ○ |
| 19. Average Length of stay | ○ | ○ | ○ | ○ | ○ |
| 20. Total Nursing Hours per Inpatient Stay | ○ | ○ | ○ | ○ | ○ |
| 21. Incidence of Adverse Events | ○ | ○ | ○ | ○ | ○ |
| 22. Mortality Rate | ○ | ○ | ○ | ○ | ○ |
| 24. Nurse Satisfaction | ○ | ○ | ○ | ○ | ○ |
| 25. Patient Satisfaction | ○ | ○ | ○ | ○ | ○ |
| 26. Nurse Turnover Rate | ○ | ○ | ○ | ○ | ○ |

1. Revision suggestions

Thanks again for your help.
